# Are undocumented migrants’ entitlements and barriers to healthcare a public health challenge for the European Union?

**DOI:** 10.1186/s40985-016-0026-3

**Published:** 2016-10-03

**Authors:** Elisabetta De Vito, Chiara de Waure, Maria Lucia Specchia, Paolo Parente, Elena Azzolini, Emanuela Maria Frisicale, Marcella Favale, Adele Anna Teleman, Walter Ricciardi

**Affiliations:** 1 Department of Human, Social and Health Sciences, University of Cassino and Southern Lazio, Campus Folcara, Via Sant’Angelo, 03043 Cassino, FR Italy; 2 Centre for Global Health Research and Studies, Catholic University of Sacred Heart, Rome, Italy; 3 Department of Public Health, Catholic University of Sacred Heart, Rome, Italy; 4 Italian National Institute of Health, Rome, Italy

**Keywords:** Undocumented migrants, Migrant health, Barriers, Access to healthcare

## Abstract

Undocumented migrants (UMs) are at higher risk for health problems because of their irregular status and the consequences of economic and social marginalization. Moreover, the emergent reality of undocumented migration in Europe calls for action in the field of management of UM’s health demands as their access to health services has become a sensitive political and social issue. In this light, this paper aims to address UMs’ entitlement and barriers to healthcare and related policies citing evidence from peer-reviewed and grey literature concerning people living in a country within the European Union without the legal right to be/remain in the destination country. A systematic review was performed using several databases and websites, and a total of 54 publications in English, with full text available, were taken into consideration.

Between 2000 and 2015, Europe hosted the second largest number of international migrants (20 million, 1.3 million per year) after Asia. Even though there is limited evidence specifically focused on UMs’ health, it is possible to state that infectious diseases, chronic illnesses, mental disorders, maternal-child conditions, dental issues, acute illnesses and injuries are the most common pathologies. In most cases across Europe, UMs have access only to emergency care. Even in countries where they are fully entitled to healthcare, formal and informal barriers hinder them from being or feeling entitled to this right. Socio-cultural barriers, such as language and communication problems, lack of formal and informal social and healthcare networks and lack of knowledge about the healthcare system and about informal networks of healthcare professionals are all common impediments. From the healthcare providers’ perspective, there can be difficulties in providing appropriate care and in dealing with cultural and language barriers and false identification. Communication strategies play a central role in addressing the inequalities in access to healthcare services, and the definition and provision of specific training, focused on UMs’ health needs, would be desirable.

Improving access to healthcare for UMs is an urgent priority since the lack of access is proven to have serious consequences for UMs’ health and wellbeing. Notwithstanding, few available examples of policies and best practices aimed at overcoming barriers in the delivery of healthcare to UMs are available.

## Background

The number of international migrants worldwide has been growing, with Europe having the second largest number of international migrants (1.3 million per year) [1]. Migration may be considered to be a social, political and health challenge because of the need to provide everyone with access to quality health services according to the concept of universal health coverage [2] and in the light of the resolution on the “health of migrants” endorsed in 2008 by the Sixty-first World Health Assembly (WHA) of the World Health Organization (WHO). The latter has brought attention to the need to provide migrants with equitable access to health promotion, disease prevention and care [3]. The challenge is great, particularly with respect to undocumented migrants (UMs)—i.e. people who enter a country without the necessary documents and permits [4]—who are considered to be one of the groups at higher risk for health problems because of their irregular status [5]. Due to different migration legal frameworks in Europe, UMs are defined in several ways. The current debate on definitions favours the use of “undocumented” and “irregular” interchangeably, whereas, due to stigmatization, “illegal” is discouraged. In particular, the Health for Undocumented Migrants and Asylum seekers (HUMA) network defines UMs as: “a) persons who are planning to seek asylum but have not formally submitted an application to asylum to the national competent authorities; b) rejected asylum seekers (those asylum seekers whose application for asylum failed); c) persons whose application for residence permit/authorization to stay/family reunification is still pending (no decision has been taken by the competent national authorities) even though in some countries they are considered to be in a regular situation; d) persons whose application for residence permit/authorisation to stay/family reunification or renewal of this authorization has failed; e) over stayers of visas (e.g. tourist, student, medical reasons); f) over stayers of expired residence or work permits; g) persons who did not apply for any visa or residence permit and entered illegally [6]”.

European countries are still not able to fully account for the demographic flows of UMs. The European Commission-funded “Clandestino” Project provided estimates of UMs in 2002, 2005 and 2008 in all 27 (at that time) member states (MS). In 2008, it was estimated that from 1.9 to 3.8 million, UMs lived in the European Union (EU) [7]. Moreover, data are almost entirely absent regarding the non-EU countries. The Frontex Annual Risk Analysis Report 2015, in analysing the data for 2014, registered a general increase in most of the indicators of irregular migration flows in the EU. The increase in illegal border-crossings reached the record value of 283,532 detections, and most of the detections at the borders concerned migrants from Syria, who later applied for asylum within the EU. Detections of illegal stay in the EU reached 441,780, which represents a generally increasing trend compared to 2013 and recent reporting periods [8, 9]. Syrian nationals registered the highest number amongst those detected in 2014 (79,169 detections) representing 28 % of the total. This was followed by Eritreans (34,586) (although their number dropped compared to the last quarter), Sub-Saharan Africans (26,341) and Afghans (22,132). The top five nationalities found in illegal stay (i.e. persons detected during travelling from external border to their final destination without proper travel documentation) were Syrians (74,723), Eritreans (34,477), Moroccans (25,329), not specified (24,461) and Afghans (23,393). Illegal trans-Mediterranean boat migration to the EU has grown steadily and is not expected to decline in the near future. Tunisia, Morocco and Libya (as of 2011) were the main North African transit countries to Europe. Boat migrants mostly consisted of men 20–40 years old and tend to be poorly educated individuals [10]. However, some were refugees and asylum seekers who did not make a voluntary choice to leave their country of origin and cannot return home in safety [11] and for whom healthcare access and entitlement represents a big challenge for European Public Health, even if the different healthcare national systems provide them with other specific guarantees [11].

The UM irregular legal status is an obstacle to access basic healthcare and social services [4]. Furthermore, UMs’ physical and mental health is expected to worsen over time [5]. Because of migration patterns, demographic profile, experiences during migration, high-risk behaviours, socioeconomic conditions and limited access to health services, UMs are more vulnerable to certain communicable diseases, occupational health hazards, injuries, poor mental health and maternal and child health problems [12–15]. Furthermore, most UMs lack information about their rights to access medical services and do not seek medical help for fear of being discovered by authorities [16].

Undocumented migration is an issue of priority for EU policy as highlighted in the main European treaties (i.e. Schengen 1985, Dublin 1990, Lisbon 2009, Stockholm 2009). The Stockholm programme is a key political document laying down the priorities and guidelines for the guarantee of security and justice [17]. Nonetheless, with the sole exception of “unaccompanied minors,” it did not explicitly refer to UMs. Furthermore, it widely adopted the term “illegality” to refer to the lack of documentation of people. The control-oriented approach on irregular migration, which is based on criminalisation, return and readmission, is prevalent in the Stockholm programme and was translated into the Action Plan elaborated by the European Commission [17–19]. In fact, the key political measures adopted in the field of irregular migration were aimed at increasing the control and surveillance of the EU external borders, at enforcing the return of UMs and in establishing administrative and penal sanctions for the third parties involved in the irregular migration process. In light of this, the objective of this paper was to synthetize research findings of available academic and grey literature on the topics of UMs’ entitlement and barriers to healthcare in order to define which policies and interventions may work to improve healthcare access and delivery in the EU.

## Methods and search results

The evidence described in this paper came from peer-reviewed literature and grey literature. The literature search was performed on PubMed, Scopus, Cochrane Library, Google and the websites of the following organizations/institutions/projects/networks: Organisation for Economic Co-operation and Development (OECD); Health Evidence Network (HEN); European Observatory; EU law and other public EU documents (EUR-lex); Community Research and Development Information Service (CORDIS); Statistical Office of the European Union (Eurostat); Evaluating the Impact of Structural Policies on Health Inequalities and their Social Determinants, and Fostering Change (SOPHIE); Migrant Integration Policy Index (MIPEX); Platform for International Cooperation on Undocumented Migrants (PICUM) and HUMA Network. A hand search of references of selected articles was also performed. Studies were considered eligible for inclusion if they were in English, had full text available and referred to the WHO European Region countries and to UMs (defined as people that do not have a legal right to be/remain in the destination country). The review was focused on entitlement to healthcare and healthcare delivery; barriers to healthcare access and policies tailored to UMs and health professionals entrusted to care for them.

After screening of titles and abstracts and after the assessment of the full text of potential eligible studies, 54 papers focused on entitlement, barriers and policy were eventually included in the review.

## Discussion

### Healthcare for UMs: entitlement and barriers

The International Covenant on Economic, Social and Cultural Rights [20] and The Charter of Fundamental Rights of the European Union [21] ratified the right of everyone to healthcare as a basic human right regardless of one’s administrative status. Despite this, a conflict between immigration policies and the human right to health is still in place. According to literature, the entitlement to have access to health services defined by the main international treaties is not yet fully respected and not all countries are working on its implementation [22–26]. In particular, most countries provide UMs with access only to emergency care and/or sometimes to certain services for specific conditions (e.g. infectious diseases) or subgroups (e.g. pregnant women, children) [7, 12]. According to MIPEX, Italy is the leading example in ensuring UMs the entitlement to healthcare [27].

The definition of a proper logical framework in order to provide a precise analysis of the state of the art in UMs’ healthcare accesses, barriers and entitlements has been structured through a research focused on both perspectives of providers and UMs themselves.

From the perspective of both professionals and UMs, regardless of country-specific entitlement to healthcare, evidence suggests that there are further formal and informal barriers in the access to healthcare by UMs. One of the most relevant formal barriers is the reporting of UMs to the legal authorities. In some countries, such as Sweden, Slovenia, the UK, Croatia and Germany, healthcare providers are required to report UMs, whereas this is forbidden in the Czech Republic, Denmark, France, Italy, Norway, Portugal, Spain and the Netherlands. In some countries, legal action can be taken against health professionals who provide care to UMs [27]. In other cases, it is left to the discretion of health professionals as to whether or not they agree to accept UMs as patients [28, 29].

Moreover, healthcare professionals face other challenges in caring for UMs, i.e. language barriers, concerns about false identification and uncertainty regarding the rules they are required to follow [30]. The lack of knowledge of UMs’ entitlement to care and complex and time-consuming paperwork represent two of the most important barriers [31]. Furthermore, difficulties in continuity of care occur even in countries where UMs are guaranteed full rights to healthcare access [32]. Resolving legal issues surrounding the patient’s irregular status can also delay care [28, 29, 33]. Lastly, professional guidelines and training initiatives aimed at coping with cultural issues are not commonly implemented in daily practice [34].

Several barriers that concern the UMs themselves were also identified. These include the following: lack of knowledge about entitlement to healthcare, lack of knowledge about the healthcare system and about informal networks of healthcare professionals, fear of being reported to the police, a sense of shame, fear of stigma, economic constraints, language and communication problems, religious practices, customs and taboos and a lack of formal and informal social and healthcare network [35–46]. As a consequence, self-treatment is a popular way for migrants to solve their health issues as well as inappropriate use of accident and emergency departments [47, 48].

### Policy recommendations

According to the results of the review, governments and policymakers should:

- Grant UMs the same entitlements to healthcare given to other residents. This is the most important challenge faced by national governments [49]. Several non-governmental organizations have lobbied for this in order to avert violating human rights [50].
- Regulate, design and adopt national policy directives. International treaties state that immediate treatment should never be withheld for any reason. However, in the real world, the lack of official national policies sometimes shifts the responsibility onto health professionals to determine who is entitled to care [33, 51].
- Strengthen collaborations at the European level. MacFarlane et al. [51] emphasized that European collaborations are necessary to identify strategies to overcome barriers and to develop culturally and linguistically appropriate healthcare systems. The management of cross-cultural communication in healthcare consultations is one of the priorities that must be addressed for future strategies for reducing inequalities in healthcare access.
- Reframe education in health science. According to Hollings et al. [52], the establishment and delivery of targeted training modules for health professionals focused on migrants would be necessary as well as interpreting services or cultural mediators [28–30].

Additionally, future planning should specifically target the following: improvement of data collection, provision of information to migrants on health problems and services and interventions aimed at modifying UMs’ care-seeking behaviour and at increasing UMs’ health literacy [52].

Evidence from Europe also conveyed key points for good management of UMs’ healthcare [44, 48, 51, 52]. Such recommendations include the following: organizational flexibility with sufficient time and resources and individualization of care, availability and quality of professional interpreting services, networking with families and social services, strengthening of interdisciplinary collaboration, implementation of mobile health units, supporting the role of non-governmental organizations, improvement of cultural awareness of health providers, development and diffusion of instructive and informative material for migrants about the healthcare system, establishment of positive relationships between staff and patients, provision of clearer information and guidelines to health providers on what type of care migrant groups are entitled to, the education and the empowerment of health professionals and students and a proper allocation of resources.

In this respect, Belgium and Scotland have already released specific recommendations in order to address migrants’ health.

In Belgium, the “ETHEALTH”—Ethnicity and Health—expert group delivered the following recommendations [53]:

- Ensuring a clear framework of reimbursement and the application of the legislation on Urgent Medical Aid
- The delivery of a voucher entitling UMs to request assistance
- Extending the use of the “medical card” to all UMs entitling them to urgent healthcare
- Diversification of health professionals and health services available to treat migrants
- Provision of a temporary residence permit for UMs affected by infectious diseases in order to assure a full course of treatment [27, 32]

Scotland recently published a policy on ethnicity and health, named as “Fair for All: Working Together Towards Culturally-Competent Service” and calling for coding and data linkage of existing health information systems; analysis of social/economic context, risk factor patterns and prevalence of major health problems and assessment of health and social care services quality [54, 55]. Subsequently, the following practical initiatives were undertaken: provision of free of charge interpreting, translation, cultural and religious services (e.g. food meeting patients’ needs) and training programmes to better address minority groups.

### Limitations

Although this study relied on an extensive systematic literature review, a quality assessment of papers included was not performed due to the heterogeneity of study designs and the absence of validated and shared tools to make this evaluation. Furthermore, the literature review did not provide evidence about the impact of policies for reducing inequalities on accessibility and quality of healthcare.

## Conclusions

There are considerable differences in the entitlement to access healthcare for UMs across Europe. According to the literature, European countries are still dealing with the challenge of fully implementing the main international treaties in order to guarantee the right to health for everyone.

Besides the differences in entitlement related to the health and social care systems, there are formal and informal barriers in access to healthcare from the point of view of both UMs and health professionals.

Formal barriers, deriving from policy framework, often prevent UMs from seeking healthcare and accessing healthcare services leading them to look for alternative strategies. According to the evidence, cultural and language barriers and differences in religious practices and customs represent an obstacle to accessing healthcare and to receiving appropriate care. Information, education and communication activities and strong networking with other social services are required both at institutional and local levels to tackle cultural and language barriers.

UMs are likely to not receive adequate healthcare and to miss out on important health services (such as maternal and child primary care or infectious disease prevention), which represents a failure for the health system [56, 57]. This is a crucial point not only from an economic point of view—because it leads to avoidable use of emergency care—but also with respect to equity and quality in healthcare.

Only a precise health policy plan will be able to overcome the above-mentioned criticalities. In light of this, the lack of demographic and health data on UMs negatively impacts the assessment of their health needs and the priority setting process.

Notwithstanding the general agreement about the need for the best practices and recommendations specifically targeted to UMs, it is extremely difficult to find examples of this in literature. From a general point of view, different strategies may be pursued in order to improve access to healthcare for UMs and should address the need to overcome formal and informal barriers from the perspective of both UMs and professionals. According to literature, future strategies in reducing inequality in healthcare access by UMs need to pay attention to providing thorough and transparent information and to communication strategies. The latter should be focused both on the right to health and on the interaction between UMs and health providers. Healthcare providers should be made aware of cultural differences [29, 33, 34, 55] and should dedicate more time to listening to and considering the needs of patients in order to cope with cultural and language barriers [58].

Dedicated communication services (i.e. cultural mediators, interpreters) would be useful in order to promote an inclusive and culturally sensitive health system. Researchers and health system experts should provide and share evidence in this field with the aim to support decision makers in policy development and monitoring.

Lastly, research on UMs’ health and current social and legal situations should be strengthened in order to foster equity in access and quality of healthcare. In fact, the scant evidence available may determine the lack of public awareness and a misestimation of UMs’ health problems and needs. Research should also be encouraged in the field of monitoring and assessing the impact of policies including the development of specific tools and indicators. All these initiatives must rely on a strong intersectoral approach and cross-border cooperation.

## Abbreviations

EU: European Union
HUMA: Health for Undocumented Migrants and Asylum seekers
MIPEX: Migrant Integration Policy Index
UM: Undocumented migrant
WHA: World Health Assembly

## References

[1] Nations U (2016). Department of Economic and Social Affairs, population division. International migration report 2015.

[2] WHO. Global coalition calls for acceleration of access to universal health coverage (http://www.who.int/universal_health_coverage/en/. Accessed 21 July 2015).

[3] Sixty-first world health assembly. Health of migrants. WHA61.17 (http://apps.who.int/iris/bitstream/10665/23569/1/A61_REC1-en.pdf. Accessed 21 July 2015).

[4] UNESCO. Migrant/migration (http://www.unesco.org/new/en/social-and-human-sciences/themes/international-migration/glossary/migrant/. Accessed 21 July 2015).

[5] Terminski B (2013). Realizing the right to health of undocumented immigrants in Europe: legal and social challenges.

[6] Access to healthcare for undocumented migrants and asylum seekers in 10 EU countries: law and practice http://www.episouth.org/doc/r_documents/Rapport_huma-network.pdf. Accessed 21 July 2015.

[7] PICUM Platform for International Cooperation on Undocumented Migrants (2007). Access to healthcare for undocumented migrants in Europe.

[8] FRONTEX. Annual risk analysis 2015 (www.frontex.europa.eu. Accessed 21 July 2015).

[9] Quarterly FRAN (2015). Quarter 3, July–September 2014.

[10] Kassar H, Dourgnon P (2014). The big crossing: illegal boat migrants in the Mediterranean. Eur J Public Health.

[11] Bradby H, Humphris R, Newall D, Phillimore J (2015). Public health aspects of migrant health: a review of the evidence on health status for refugees and asylum seekers in the European Region.

[12] Dias S, Gama A, Cortes M, Sousa B (2011). Healthcare-seeking patterns among immigrants in Portugal. Health Soc Care Commun.

[13] Sandhu S, Bjerre NV, Dauvrin M, Dias S, Gaddini A, Greacen T (2012). Experiences with treating immigrants: a qualitative study in mental health services across 16 European countries. Soc Psychiatry Psychiatr Epidemiol.

[14] Schoevers MA, Loeffen MJ, van den Muijsenbergh ME, Lagro-Janssen AL (2010). Healthcare utilisation and problems in accessing healthcare of female undocumented immigrants in the Netherlands. Int J Public Health.

[15] Almeida LM, Caldas J, Ayres-de-Campos D, Salcedo-Barrientos D, Dias S (2013). Maternal healthcare in migrants: a systematic review. Matern Child Health J.

[16] Mladovsky P, Ingleby D, McKee M, Rechel B (2012). Good practices in migrant health: the European experience. Clin Med.

[17] Council of the European Union, the Stockholm programme: an open and secure Europe serving and protecting citizens, 5731/10, Brussels, 3 March 2010.

[18] PICUM Platform for International Cooperation on Undocumented Migrants (http://picum.org/en/press-room/guidelines-for-journalists/. Accessed 21 July 2015).

[19] Merlino M, Parkin J. Irregular migration in Europe: EU policies and the fundamental rights gap- 2011 ETUC- CESP report.

[20] International Covenant on Economic, Social and Cultural Rights or on the website of the Office of the United Nations High Commissioner for Human Rights (www.ohchr.org). Accessed 26 July 2016.

[21] Commission of the European Communities (2000a) Communication of the commission on the legal nature of the charter of fundamental rights of the European Union. COM(2000) 644 final, 11 October 2000, available at http://eur-lex.europa.eu/legal-content/EN/TXT/?uri=CELEX%3A52000DC0644. Accessed 26 July 2016

[22] Network HUMA (2011). Are undocumented migrants and asylum seekers entitled to access healthcare in the EU? A comparative overview in 16 countries.

[23] Mylius M, Frewer A (2015). Access to healthcare for undocumented migrants with communicable diseases in Germany: a quantitative study. Eur J Public Health.

[24] Poduval S, Howard N, Jones L, Murwill P, McKee M, Legido-Quigley H (2015). Experiences among undocumented migrants accessing primary care in the United kingdom: a qualitative study. Int J Health Serv.

[25] Woodward A, Howard N, Wolffers I (2014). Health and access to care for undocumented migrants living in the European Union: a scoping review. Health Policy Plan.

[26] Suess A, Ruiz Perez I, Ruiz Azarola A, March Cerda JC (2014). The right of access to healthcare for undocumented migrants: a revision of comparative analysis in the European context. Eur J Public Health.

[27] MIPEX Migrant Integration Policy Index (http://www.mipex.eu. Accessed 21 July 2015).

[28] Jensen NK, Norredam M, Draebel T, Bogic M, Priebe S, Krasnik A (2011). Providing medical care for undocumented migrants in Denmark: what are the challenges for health professionals?. BMC Health Serv Res.

[29] Grit K, den Otter JJ, Spreij A (2012). Access to healthcare for undocumented migrants: a comparative policy analysis of England and the Netherlands. J Health Polit Policy Law.

[30] Biswas D, Kristiansen M, Krasnik A, Norredam M (2011). Access to healthcare and alternative health-seeking strategies among undocumented migrants in Denmark. BMC Public Health.

[31] Goossens MC, Depoorter AM (2011). Contacts between general practitioners and migrants without a residence permit and the use of “urgent” medical care. Scand J Public Health.

[32] Dauvrin M, Lorant V, Sandhu S, Devillé W, Dia H, Dias S, Gaddini A (2012). Healthcare for irregular migrants: pragmatism across Europe. A qualitative study. BMC Research Notes.

[33] Gray BH, van Ginneken E (2012). Healthcare for undocumented migrants: European approaches. Issue Brief (Commonw Fund).

[34] Van den Muijsenbergh M, van Weel-Baumgarten E, Burns N, O’Donnell C, Mair F, Spiegel W (2014). Communication in cross-cultural consultations in primary care in Europe: the case for improvement. The rationale for the RESTORE FP 7 project. Prim Healthcare Res Dev.

[35] Costa D, Matanov A, Canavan R, Gabor E, Greacen T, Vondráčková P (2014). Factors associated with quality of services for marginalized groups with mental health problems in 14 European countries. BMC Health Serv Res.

[36] Cuadra CB (2011). Right of access to healthcare for undocumented migrants in EU: a comparative study of national policies. Eur J Public Health.

[37] Devillé W, Greacen T, Bogic M, Dauvrin M, Dias S, Gaddini A (2011). Healthcare for immigrants in Europe: is there still consensus among country experts about principles of good practice? A Delphi study. BMC Public Health.

[38] Fakoya I, Reynolds R, Caswell G, Shiripinda I (2008). Barriers to HIV testing for migrant black Africans in Western Europe. HIV Med.

[39] Huffman SA, Veen J, Hennink MM, McFarland DA (2012). Exploitation, vulnerability to tuberculosis and access to treatment among Uzbek labor migrants in Kazakhstan. Soc Sci Med.

[40] Kluge U, Bogic M, Devillé W, Greacen T, Dauvrin M, Dias S (2012). Health services and the treatment of immigrants: data on service use, interpreting services and immigrant staff members in services across Europe. Eur Psychiatry.

[41] Priebe S, Matanov A, Barros H, Canavan R, Gabor E, Greacen T (2012). Mental health-care provision for marginalized groups across Europe: findings from the PROMO study. Eur J Public Health.

[42] Priebe S, Matanov A, Schor R, Straßmayr C, Barros H, Barry MM (2012). Good practice in mental healthcare for socially marginalised groups in Europe: a qualitative study of expert views in 14 countries. BMC Public Health.

[43] Lindert J, Schouler-Ocak M, Heinz A, Priebe S (2008). Mental health, healthcare utilisation of migrants in Europe. Eur Psychiatry.

[44] Priebe S, Sandhu S, Dias S, Gaddini A, Greacen T, Ioannidis E (2011). Good practice in healthcare for migrants: views and experiences of care professionals in 16 European countries. BMC Public Health.

[45] Teunissen E, Sherally J, van den Muijsenbergh M, Dowrick C, van Weel-Baumgarten E, van Weel C (2014). Mental health problems of undocumented migrants (UMs) in the Netherlands: a qualitative exploration of help-seeking behaviour and experiences with primary care. BMJ Open.

[46] Wolff H, Epiney M, Lourenco AP, Costanza MC, Delieutraz-Marchand J, Andreoli N (2008). Undocumented migrants lack access to pregnancy care and prevention. BMC Public Health.

[47] Buja A, Fusco M, Furlan P, Bertoncello C, Baldovin T, Casale P (2014). Characteristics, processes, management and outcome of accesses to accident and emergency departments by citizenship. Int J Public Health.

[48] Mukomel V (2013). Integration of migrants: Russian federation, CARIM-East RR 2013/02, Robert Schuman centre for advanced studies.

[49] Mladovsky P, Rechel B, Ingleby D, McKee M (2012). Responding to diversity: an exploratory study of migrant health policies in Europe. Health Policy.

[50] Hellgren Z (2014). Negotiating the boundaries of social membership: undocumented migrant claims-making in Sweden and Spain. J Ethn Migr Stud.

[51] MacFarlane A, O’Reilly-de Brún M, de Brún T, Dowrick C, O’Donnell C, Mair F (2014). Healthcare for migrants, participatory health research and implementation science—better health policy and practice through inclusion. The RESTORE project. Eur J Gen Pract.

[52] Hollings J, Samuilova M, Petrova-Benedict R (2012). Health, migration and border management: analysis and capacity-building at Europe’s borders. Int J Public Health.

[53] Dauvrin M, Derluyn I, Coune I, Verrept H, Lorant V. Towards fair health policies for migrants and ethnic minorities: the case-study of ETHEALTH in Belgium. BMC PUBLIC HEALTH. 2012;12. 10.1186/1471-2458-12-726 PMC3520724 22938597

[54] Scottish Executive (2002). Fair for all: working together towards culturally-competent services.

[55] Bhopal RS (2012). The quest for culturally sensitive health-care systems in Scotland: insights for a multi-ethnic Europe. J Public Health.

[56] Higginbottom G, Reime B, Bharj K, Chowbey P, Ertan K, Foster-Boucher C (2013). Migration and maternity: insights of context, health policy, and research evidence on experiences and outcomes from a three country preliminary study across Germany, Canada, and the United Kingdom. Healthcare Women Int.

[57] Degni F, Suominen S, Esse B (2012). Communication and cultural issues in providing reproductive healthcare to immigrant women: healthcare providers’ experiences in meeting the needs of [corrected] Somali women living in Finland. J Immigrant Minority Health.

[58] Jensen NK, Nielsen SS, Krasnik A (2011). Expert opinion on best practices “in the delivery of healthcare services to immigrants in Denmark”. Dan Med Bull.

